# Staying healthy “under the sheets”: Inuit youth experiences of access to sexual and reproductive health and rights in Arviat, Nunavut, Canada

**DOI:** 10.3402/ijch.v75.31812

**Published:** 2016-12-09

**Authors:** Gregory J Corosky, Astrid Blystad

**Affiliations:** Faculty of Medicine and Dentistry, Centre for International Health, Department of Global Public Health and Primary Care, University of Bergen, Bergen, Norway

**Keywords:** sexual health, reproductive health, health and rights, access to care, Inuit youth, prevention, community-driven, youth health, structural factors, structural barriers

## Abstract

**Background:**

Inuit youth are reported to experience considerably worse sexual and reproductive health and rights (SRHR) outcomes than Canadian youth in general, as evidenced through public health data on sexually transmitted infections, unintended young pregnancies and rates of sexual violence in Nunavut compared to national averages. Existing literature on Inuit SRHR has identified the impact of westernization and colonialism on health outcomes, though gaps remain in addressing youth- and community-specific experiences of SRHR.

**Objective:**

This study aims to generate youth-focused evidence on experiences of SRHR relating to access to care in Arviat in order to better inform locally authored interventions geared towards improving youth SRHR.

**Design:**

The *Piliriqatigiinniq* Partnership Community Health Research Model (PRM) developed by the Qaujigiartiit Health Research Centre was followed to generate data on youth experiences of SRHR support access in Arviat. In-depth interviews were conducted with 9 male youth (ages 17–22 years), 10 female youth (ages 16–22 years) and 6 community leaders (aged 25+). Snowball sampling was used to engage informants, and data analysis followed an approach similar to conventional content analysis, where emphasis was placed on “immersion and crystallization” of data, corresponding to the Inuit concept of *Iqqaumaqatigiinniq* in the PRM. Findings were continuously checked with community members in Arviat during the analysis phase, and their feedback was incorporated into the report.

**Results:**

Youth in Arviat were found to face significant barriers to SRHR care and support. Three major themes emerged as important factors conditioning youth access to SRHR resources in the community: trust of support workers in the community; stigma/taboos surrounding SRHR topics; and feelings of powerlessness impeding female and lesbian/gay/bisexual/transgender/queer youth in particular from accessing care.

**Conclusions:**

The locally specific ways these themes emerged revealed important structural factors at play in the community, which seem to persistently work against youths’ abilities to achieve good SRHR outcomes. To address poor micro-level health outcomes in Arviat, it thus appears that locally authored programming must take into account broader structural factors at the root of SRHR access barriers.

## Lexicon of terms

- *SRHR (sexual and reproductive health and rights)*: “encompass the right of all individuals to make decisions concerning their sexual activity and reproduction free from discrimination, coercion, and violence … SRHR includes the right of all persons to: Seek, receive, and impart information related to sexuality; Receive sexuality education; Have respect for bodily integrity; Choose their partner; Decide to be sexually active or not; Have consensual sexual relations; Have consensual marriage; Decide whether or not, and when, to have children; and Pursue a satisfying, safe, and pleasurable sexual life” (1, p. 1)
- *LGBTQ*: Lesbian/gay/bisexual/transgender/queer
- *Hamlet*: A small settlement
- *Arviarmmiut*: People from Arviat (singular: *Arviarmmiuq*)
- *Nunavummiut*: People from Nunavut (singular: *Nunavummiuq*)
- Qaujigiartiit Health Research Centre: A Nunavut-based research centre that developed the Piliriqatigiinniq Partnership Community Health Research Model (PRM)
- *Piliriqatigiinniq*: Working in a collaborative way for the common good (H&T Sr. 1)

Inuit youth in Nunavut experience drastically poorer sexual and reproductive health and rights (SRHR) outcomes than their Canadian peers. High rates of sexually transmitted infections (STIs) are a “major ongoing concern” for the territory, with population health data from 2014 indicating transmission rates over 10 times the national average for chlamydia, gonorrhoea and syphilis (Supplementary file 1) (2, p. 33). Youth between the ages of 15 and 24 constitute the majority of new chlamydial and gonorrhoeal infections, and syphilis rates began rapidly increasing in 2012 following an outbreak (Supplementary file 1) (2, p. 3). The teenage (aged 14–19 years) pregnancy rate in Nunavut is over five times the national average (3, p. 6), with Arviat reporting to be the community with the highest birth rate in Canada, at 35 births per 1,000 people (compared to the national average of 10.3 per 1,000) (4). A disturbing incidence of 52% of women and 22% of men are found to have experienced “severe sexual abuse during childhood,” and Nunavut's rate of sexual assault crimes is over nine times the national average (with children under 18 in Nunavut being “10 times more likely than their Canadian peers to experience sexual violations”) (3, p. 7).

These health and social challenges persist under external structural factors, generated by a still prevailing colonialism, that strongly work against good SRHR outcomes for Inuit youth. Several authors have noted the effects of widespread poverty, female disempowerment, intergenerational trauma, systemic breakdown of familial relationships and a lack of culturally sensitive care on Inuit health and Indigenous well-being in general (5–10).

## SRHR in Arviat

The study on which this article is based focuses on the hamlet of Arviat: a fly-in community of some 2,600 people located on the northwest coast of Hudson Bay in Nunavut (see Supplementary file 2) (11). Arviat has a very high youth population, with nearly 40% of the demographic being under the age of 15 in 2006 (compared with 18% of the Canadian population for the same year) (11). Ninety-two percent of Arviat's population is Inuit, and Inuktitut remains widely spoken among both youth and elders (11). There is a Wellness Centre that runs health promotion programming and a Health Centre with a medical clinic. The Health Centre has permanent nurses, community and public health workers, and a drop-in clinic. Patients requiring urgent care often rely on air travel to Winnipeg because there is no permanent physician on staff in Arviat.

Arviat faces substantial SRHR challenges today. Nonetheless, community programming focusing on adolescent SRHR has not existed in a sustained or systematic manner to date (Conversation with: Shirley Tagalik, September 2014). There is no shelter for abused women and/or youth, and resources for lesbian/gay/bisexual/transgender/queer (LGBTQ) youth are largely non-existent. External structural factors such as poverty and ongoing historical trauma further exacerbate SRHR outcomes for youth in the hamlet. While adolescent SRHR is a domain of increasing attention for public health researchers globally, important gaps remain in addressing these critical issues for Inuit youth.

## Aims

This study aims to contribute to a broader body of youth-focused evidence on SRHR to help inform more effective and relevant community-driven programming. Through in-depth interviews with youth and community leaders, the study attempts to explore experiences of SRHR among Inuit youth in Arviat, including access barriers to support, community leader perceptions of youth SRHR experiences and ways to make youth SRHR programming more effective. It does not attempt to set out specific intervention strategies for Arviat or other northern communities, but aims to provide youth-generated evidence for effective community-authored programming. Its findings reflect the experiences as presented by the youth and community leaders who were interviewed in Arviat. A rigorous application of qualitative methods, however, may produce findings that point towards key issues pertaining to youth SRHR improvement in Nunavut more broadly.

## Brief literature review

Existing literature on SRHR among Inuit youth is useful but limited. Steenbeek et al. (10) and Healey (12) have outlined how current behaviours and health outcomes are shaped by precontact cultural practices and ongoing colonial histories. Healey's qualitative study, based on in-depth interviews, highlights westernization “fractured family relationships and communication about sexuality,” leading to a rupture in reproductive health knowledge being passed from one generation to the next (12, p. 136). The study is conducted “within an indigenous knowledge framework with a focus on Inuit epistemology and methodology, specifically the *Piliriqatigiinniq* Partnership Community Health Research Model” (12, pp. 134–5). Steenbeek et al. also discuss the breakdown of reproductive health teaching within families, and further cite the historic cultural importance placed on childbearing to explain high levels of sexual autonomy among youth today (10, p. 533). The authors claim that youth remain sexually autonomous at present, but since parents “no longer feel competent” teaching reproductive health to their children, condom use is infrequent. This has led to a drastic increase in STI transmission (10, p. 533). Youth perspectives on SRHR are not directly focused on in Steenbeek et al.s or Healey's work, and may provide further insight alongside findings from Inuit parents.

Cole (13) and the Government of Nunavut (GN) (14) moreover conducted studies on SRHR in Nunavut. Cole collected data through written surveys from youth informants, and found high self-reported risky sexual behaviour and a general consensus on inadequate sexual health education in schools (13, p. 272). The GN's 2013 study was based on discussions between students and community health workers, and found varied experiences with both the quantity and quality of sexual health education in Nunavut (14, p. 3,5). Youth were found to feel most comfortable talking to health care workers (nurses, doctors and other health workers), followed by people with relevant lived experiences, and finally by family members, on matters regarding SRHR (14, p. 6–7).

This study seeks to help bridge some of the gaps in the existing literature by engaging youth who both attend and do not attend school in in-depth interviews, which is important given the large number of non-attenders in the territory (54% of 25–64 year-olds in Nunavut have completed at least high school in comparison to the national average of 89% for the same age bracket) (15). Community leaders are also interviewed to gain multifaceted perspectives on youth sexual health in the community. By recognizing the impact of colonial legacies of westernization as outlined by Steenbeek et al. and Healey, this study aims to add to the current literature on Inuit adolescent sexual health in a decolonizing, community-focused and youth-driven manner.

This study is also guided by the tenets of the *Piliriqatigiinniq* Partnership Community Health Research Model (PRM) used by Healey. The PRM was developed by the Qaujigiartiit Health Research Centre “in response to the community-identified need for health research that explores topics of concern to Nunavummiut, and is collected, analysed and disseminated in a holistic and collaborative way” (16, p. 10). The model is built on Inuit “relational epistemology:” “a foundation for knowing … based on the formulation of relationships among members of the community of knowers” (16, p. 3). The PRM is made up of four pillars derived from Inuit cultural knowledge: *Inuuqatigiitsiarniq* (“being respectful of all people”), *Unikkaaqatigiinniq* (“story-telling”), *Pittiarniq* (“being kind and good”), and *Iqqaumaqatigiinniq* (“all things coming into one”) (16, p. 1). These principles are much deeper and more complex than can be fully described here (16, p. 10), not least since we are non-Inuit authors. However, following the PRM for practical guidance is a vital aspect of our attempt to engage in decolonizing research.

## The present study among Inuit youth in Arviat

### Study setting: a brief historic backdrop

For thousands of years, Inuit have inhabited the northern circumpolar region of the globe, covering an area from the Chukchi Peninsula of eastern Russia, across Alaska and northern Canada, to Greenland's southeast coast (17, p. 2). Nomadic land-based lifestyles were practiced by Inuit in Canada's Arctic “until just a generation ago,” when a history of European contact and Canadian federal interventionism culminated in the Inuit becoming largely dependent on the government for the first time in their long history of autonomy and self-reliance (17, p. 8; 17).

Profound changes to Inuit ways of life occurred through a process of forced relocations by the Canadian federal government initiated in the 1940s and continuing during the 1950s and the 1960s (18, p. 3). Inuit were moved from their dispersed locations onto permanent, centralized settlements created by “state planners” (19, p. 7). This shift marked a rupture from customary nomadic life which “drastically altered social environments,” and brought on “profound economic, social, and cultural changes” (20, p. 3). The Canadian federal government also began a system of residential schools for Inuit children in 1951 in an attempt to assimilate Inuit peoples into mainstream Canadian culture and life (21, pp. 26–7). Over 75% of school-aged Inuit children by 1964 attended residential schools, where “Inuit children were forbidden to speak their own language or practice any aspect of their culture in the school, dormitories, hostels and other residences,” and were “made to feel ashamed of their traditional way of life” (21, p. 24, 26). The residential school system became known to expose children to physical, sexual and emotional abuse (20, p. 27; 22, pp. 3–4).

Colonial policies have left a legacy of intergenerational trauma on Inuit individuals and communities, which continues to have negative impacts on mental, physical and social health and well-being (23, p. 409; 24). These policies have also led to broader “political, cultural, economic and social disenfranchisement” (25, p. S58), creating disproportionate structural barriers that further work against good health for Inuit. As Tagalik succinctly summarises, “ill health stems from a fundamental breakdown in the conditions that support health, either physical, socio-cultural, economic, or mental. In the case of post-colonial trauma, all of these conditions became broken for Inuit populations” (26, p. 5).

### Methods

The *Piliriqatigiinniq* Partnership Community Health Research Model (PRM) guides the study. The aforementioned four pillars of the PRM are nuanced principles of Inuit knowledge, but have been operationalized as practical guidelines for researchers collaborating with Inuit communities to include (inter alia): reflexivity of the researcher, meaningful community engagement, developing and fostering relationships, sharing and finding meaning and understanding in stories, and doing good as defined by Inuit conceptions of goodness (16, pp. 5–9). A conscious effort was made to incorporate these guidelines in specific ways throughout the research process, and collaboration with Arviarmmiut was emphasized in particular to aid in this process.

Fieldwork was carried out in Arviat for 2 months, and involved actively engaging with youth in the high school, Wellness Centre and daily life (e.g. social events at the Community Hall, a land-trip with local youth who were hunting caribou, Halloween festivities, etc.). Developing a rapport with youth across the community helped build trust, understanding and a clearer vantage point for the interviews. The target groups are Arviarmmiut youth between the ages of 15 and 24, and community leaders above the age of 24. Community leaders must have regular interaction with community youth or work with health-related services. In total, 9 male youth between 17 and 22 years, 10 female youth between 16 and 22 years, and 6 community leaders were interviewed.

The study employed snowball sampling – a method in which informants suggest other potential interview participants as the study progresses – with the initial help of the Wellness Centre and key community youth contacts. Interviews were conducted with community leaders and male youth in English, and an Arviarmmiuq woman, Martha Pingushat, conducted the female youth interviews in English and/or Inuktitut. The interviews were conducted in the research bunkhouse or at another location of the informant's choosing. They were conducted as open conversations shared in a comfortable atmosphere. Rigorous informed consent was a critical component of data collection and included a conversation about the study with verbal consent, as well as a written consent form. Informants were reminded they were not required to answer questions posed, could stop the discussion at any time and were encouraged to ask clarifying questions. Interviews were recorded and transcribed, and an Arviarmmiuq project collaborator translated those conducted in Inuktitut (after signing a confidentiality agreement).

Data analysis was an ongoing process from the beginning of the stay in Arviat, including during the interviews, and questions were continuously adapted to better suit the reality of the community youth. After the interviews were finished, transcribed and checked for verbatim transcription, they were carefully read and reflected upon alongside field notes. Bits of text were highlighted and condensed to extract codes, which were merged into categories. As the categories grew, some were consolidated with others, and broader topics began to emerge from the groups of codes. Healey notes that such a process of “immersion and crystallization” of data analysis “is analogous to the Inuit concept of *Iqqaumaqatigiinniq*;” a pillar of the PRM interpreted in this context to mean “the concept that ideas or thoughts may come into ‘one’” (12, p. 135). The central findings or themes were sent back to the community for feedback that in turn was incorporated into the analysis. A Skype conference with community leaders and youth in the hamlet was held to verbally discuss the findings and gather additional feedback.

## Results

### Trust and SRHR support

Three interrelated major themes emerged from the data, and key quotations are highlighted here to illustrate the most salient findings of the investigation. The first is the importance of trust for access to community SRHR support. Most young people stated they either rarely or never feel comfortable enough to talk to community health workers about sexual health topics, but that they have greater trust for support contacts they have a personal relationship with. When discussing whether he thinks his friends and peers feel comfortable seeking SRHR-related support at the Health Centre, one youth stated: “No. Because I learned that people don't trust easily. Unless they know the person that's working at the Health Centre they wouldn't ask.” Most community leaders highlighted trust as an important factor influencing the youths’ ability to seek support, with one leader stating, “Unless [the youth] have a family member who works for the Wellness or Health Centre, they stay away from it.” Another leader said, “The most recurrent theme that I heard is that [the youth] don't trust the people [at the Health or Wellness Centres] to maintain confidentiality, that there's gossip or information shared at those places. So they're people you don't trust to support you in a proper way.” Among youth who reported feeling comfortable accessing SRHR support, this was generally only the case after an acute onset of physical symptoms following a sexual encounter.

The transiency of support personnel was found to be an important factor influencing youths’ trust of SRHR support. Many health workers and educators are in Arviat on temporary work contracts, which was found to impede youth from building trusting relationships that emerge as an important precondition for accessing care. One youth remarked, “people at the Health Centre aren't gonna tell me what to do when I'm sick. They're not gonna help me forever, so I need to do things on my own.” Community leaders echoed the problem of transiency on trust, with one stating, “Trust is a barrier … They know we're only here for a time. So if they invest in you and they share too much with you and you leave, you're gone – they've lost you. So there's that mistrust.”

Trust was seen to be higher for support contacts that speak from first-hand experience rather than abstract or school-based information. When discussing how they could feel more comfortable learning about SRHR topics, one youth responded: “Other peoples’ experiences maybe. I think people my age would have more trust in their friend who they are close to … more relatable. More understanding.” Most young people also emphasised the ineffectiveness of learning about SRHR in school, with one youth stating, “I don't think they teach it in high school,” and another stating “I had to talk to my parents about [sexual health] because I could not understand anything at school. So I would ask my parents about stuff like that.”

### Taboos/stigma and SRHR support

The second theme highlights taboos and stigma as barriers to seeking community SRHR support.

The majority of youth indicated they never or rarely discuss SRHR with anyone, and this pervasive lack of general dialogue seems to leave youth too shy or scared to seek support. One youth, when asked why people his age may not seek support, stated: “Because you don't ask. It's something scary … inside you that doesn't want to ask, but wants to know.” Shyness was also noted to impact access to condoms. When asked if most of their peers use condoms, one youth stated,

No … most of Arviat is too shy or embarrassed to ask for condoms. [It is easy to get condoms in Arviat], but most people don't know that. Because they don't tell [the youth]. Or, it is not spoken about – the word is not spread out enough.

Another said,

[Young people] are shy. They think they're the only ones [to get an STI] so they don't want to talk about it. Scared. Maybe went under the sheets and got infections too many times … and got shy and didn't want to go back to the Health Centre.

Youth and community leaders also expressed worry about others’ reactions as a barrier to support, with many informants citing Arviat's small size and the limited SRHR-support access points as deterrents to seeking care. One community leader emphasised, “Being a small community, everyone knows everyone, and they're talking about this and that and what happened, and it's hard to live a private life.”

### Feelings of powerlessness and SRHR support

The third theme highlights feelings of powerlessness as barriers to SRHR support, and identifies young women and LGBTQ youth as particularly blocked from support due to feelings of disempowerment. Gender inequality leading to women feeling disempowered from seeking care was a prominent finding across interviews, with one community leader stating,

It's a boy's world here … So whatever the guys says [sic], you have to do. There's no real saying no, and in some cases the girls don't know how to say no to the guy; they're afraid of what would happen if they did. Because abuse is very prevalent in this community.

Another community leader echoed many female youth are taken advantage of by men, stating,

… they're young girls, and there's older boys who are now interested in them …They feel like this is a way to get what they're missing. Because a lot of them feel like no one listens to them, no one cares about them. People are always mean and hurtful, yelling at them. So if you're 14 years old and a 20 year old is now going to pay you attention and … wants to have sex with you, then you probably feel like, well, okay …. So I think that's a huge issue.

The problem young people have in reporting such situations of abuse was also noted, with one community leader explaining,

There's a lot of sexual abuse that goes on in the community … Young people know if you go and talk to a teacher, they're going to report it, and you're going to be in this system that is horrific. That doesn't get you the help you need … that further victimizes you.

In addition to girls being victims of sexual abuse, LGBTQ youth emerged as a vulnerable category of youth, as at present this group is generally unable to access SRHR support. One community leader stated,

Even though I know there are kids struggling with that, there are very few that would ever come out and admit it. There's a lot of fear around it.

Another stated,

A … stereotype is that homosexuals are bad. They’re not welcome in town …. When the government shoved religion down our throats … there was the Roman Catholic and Anglican churches … and they're not open to people who are different … they said this is black, this is white, and they don't mingle. There's no grey, there are no shades, there are no colours … When you're a homosexual, you're automatically bad.

While the majority of female youth informants voiced negative perceptions of LGBTQ topics, the majority of male youth informants showed a more supportive or ambivalent attitude towards LGBTQ topics. When asked how they would respond if a friend came out to them as gay, one youth responded: “I would tell them it is good they can tell me – it's a sign they trust me. I would just try to talk to them,” and another stated, “Gay is good. It's their own life, so I wouldn't mind … If they were upset, I'd tell them it's none of other peoples’ business. It's their lives, they can do what they want to.”

## Discussion and concluding remarks

Youth in Arviat seem to face numerous access barriers to SRHR knowledge and preventive support that would help mitigate STI transmission risk, and protect youth from sexual abuse. The main findings of trust, taboos and stigmas, and feelings of powerlessness influencing access to support are not novel in and of themselves in global SRHR literature (27–32). However, they emerge in the present investigation in locally specific ways, revealing particular challenges faced by Arviat youth in light of the community's overarching structural factors. Such factors nourish and maintain access barriers to preventive SRHR knowledge and support that would mitigate STI transmission risk, and protect youth from sexual abuse.

Lack of trust preventing youth from seeking support, for example, emerges as closely linked to the transiency of educators and health workers who do not remain in the hamlet long enough to develop the trusting rapport youth identify as a precondition to accessing care. Heavy taboos and stigmas preventing youth from seeking care are tied to confidentiality issues and the lack of choices young people have when in need of care – a challenge exacerbated by poverty and the constraints of living in a fly-in community. Feelings of powerlessness preventing access to care for female and LGBTQ youth were observed alongside pervasive sex and gender norms, which themselves were found to have been imposed upon the community through rapid colonisation.

Given the local manifestations of the themes identified in this study, these findings may aid community-driven interventions in measures to effectively address barriers to SRHR among youth in Arviat. A collaborative community focus on preventive SRHR care for adolescents does emerge as beneficial to future programming, though this strategy relies on the notion that greater awareness of sexual health topics will lead to better SRHR outcomes. In order for change to occur, overarching structural factors must be addressed alongside local programming. Steenbeek (33) notes “those who practice empowerment must continually combine the idealism of community with persisting social and economic conditions that foster fear, inequality, and poor health,” and that “altering structural power inequalities is an important facet of empowerment and health promotion, especially in STI prevention” (p. 263).

This discussion does not claim that changing local programmes will single-handedly solve poor SRHR outcomes and the structural factors perpetuating them. However, these issues will be difficult – if not impossible – to remedy unless the voice of Inuit youth, in relation to community services, is seriously taken into account. Findings on trust in particular suggest that locally grown youth-informed initiatives may prove more effective in increasing access to prevention, counselling and support than outside workers (including physicians) coming into the community on work contracts, or intervening with non-tailored programming. While a permanent physician would undoubtedly be of great benefit to the community, this addresses merely one part of the greater problem. Acute medical care is unlikely to solve the complex upstream structural factors that emerge as the most significant source of poor SRHR outcomes, and even if a physician was stationed in the community, there is no indication that youth would seek their help or support unless various access barriers were addressed.

Future research on SRHR among Inuit youth should incorporate the PRM guidelines and focus on topics identified as important by Inuit youth themselves-including overarching structural factors-with particular focus on more vulnerable subgroups such as female and LGBTQ youth in Nunavut. Studies should continue to collaborate with local stakeholders to build further evidence that can help inform locally authored preventive interventions.

## Supplementary Material

Staying healthy “under the sheets”: Inuit youth experiences of access to sexual and reproductive health and rights in Arviat, Nunavut, Canada
