# Supplementary material for: Staying healthy “under the sheets”: Inuit youth experiences of access to sexual and reproductive health and rights in Arviat, Nunavut, Canada
Source: Int J Circumpolar Health. 2016 Dec 9;75:10.3402/ijch.v75.31812. doi: 10.3402/ijch.v75.31812 (PMC5149654; doi:10.3402/ijch.v75.31812)
Supplement: Staying healthy “under the sheets”: Inuit youth experiences of access to sexual and reproductive health and rights in Arviat, Nunavut, Canada [file IJCH-75-31812-s001.pdf]

Appendix 1: Tables

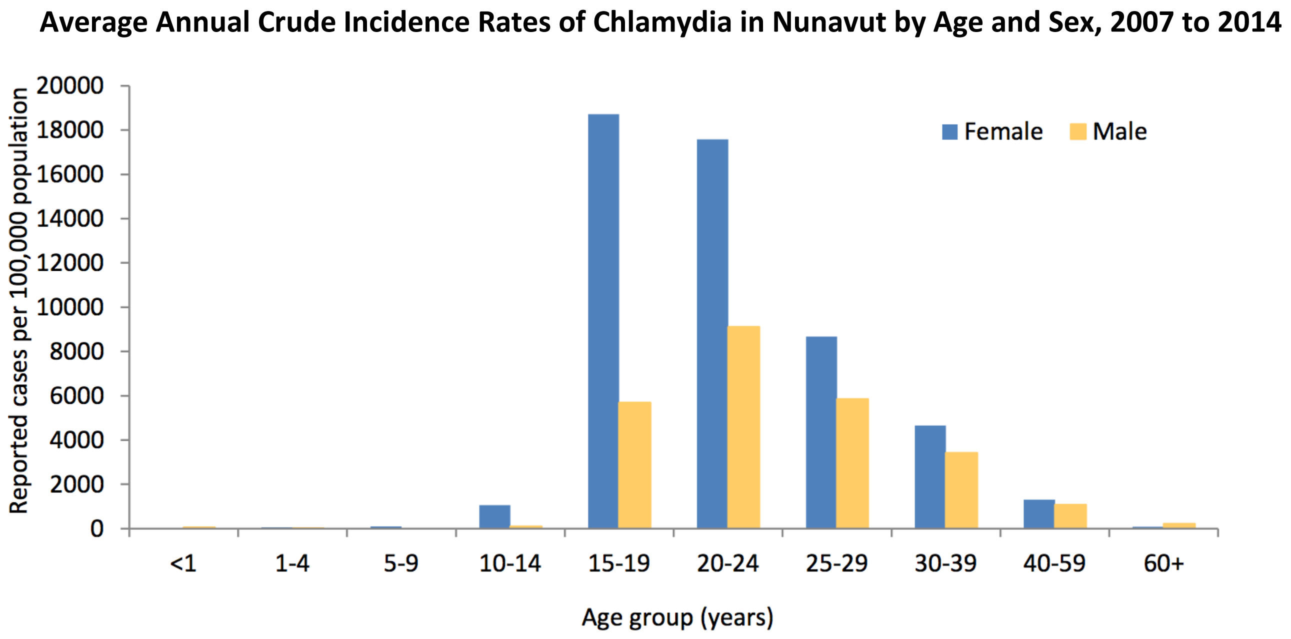

Figure 1(1, p11).

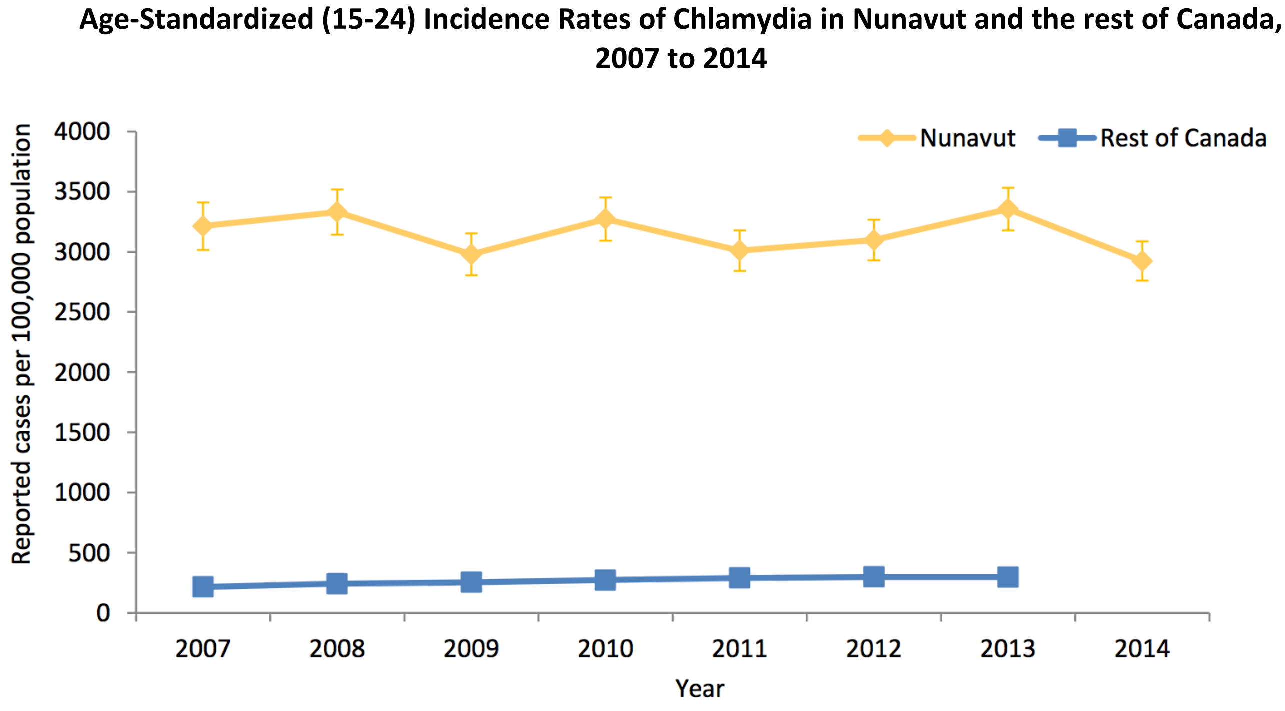

Figure 2 (1, p10).

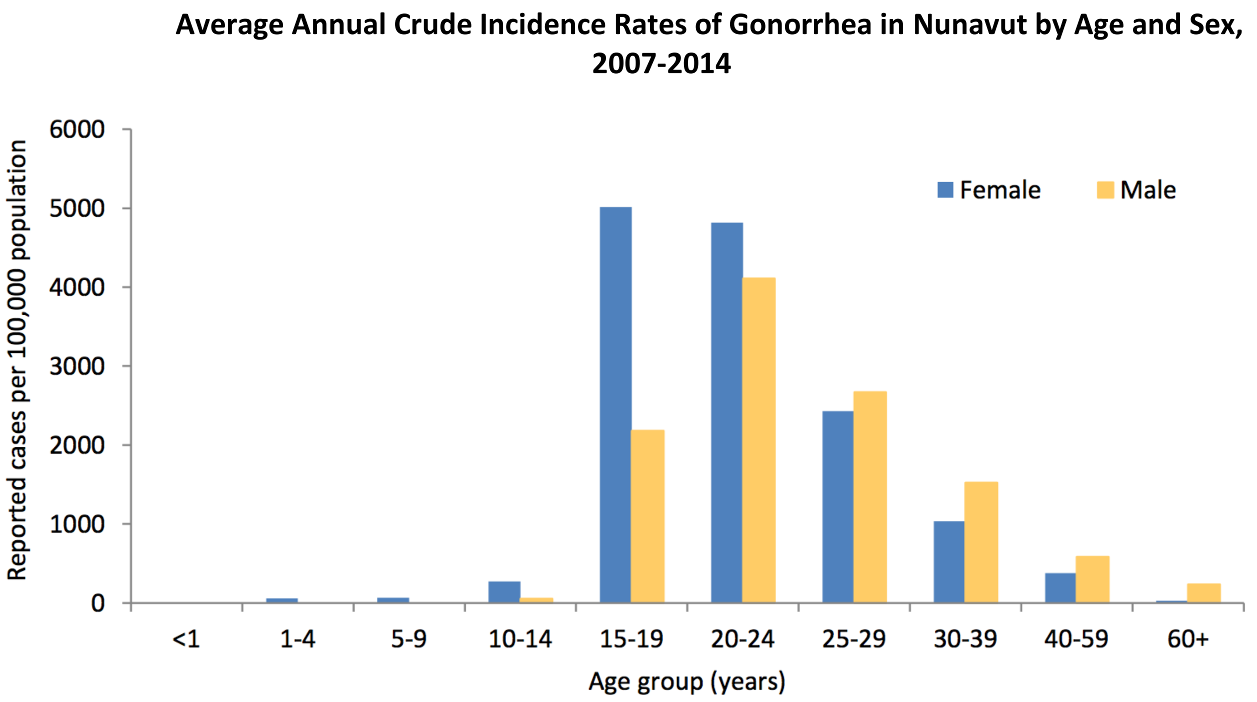

Figure 3 (1, p13).

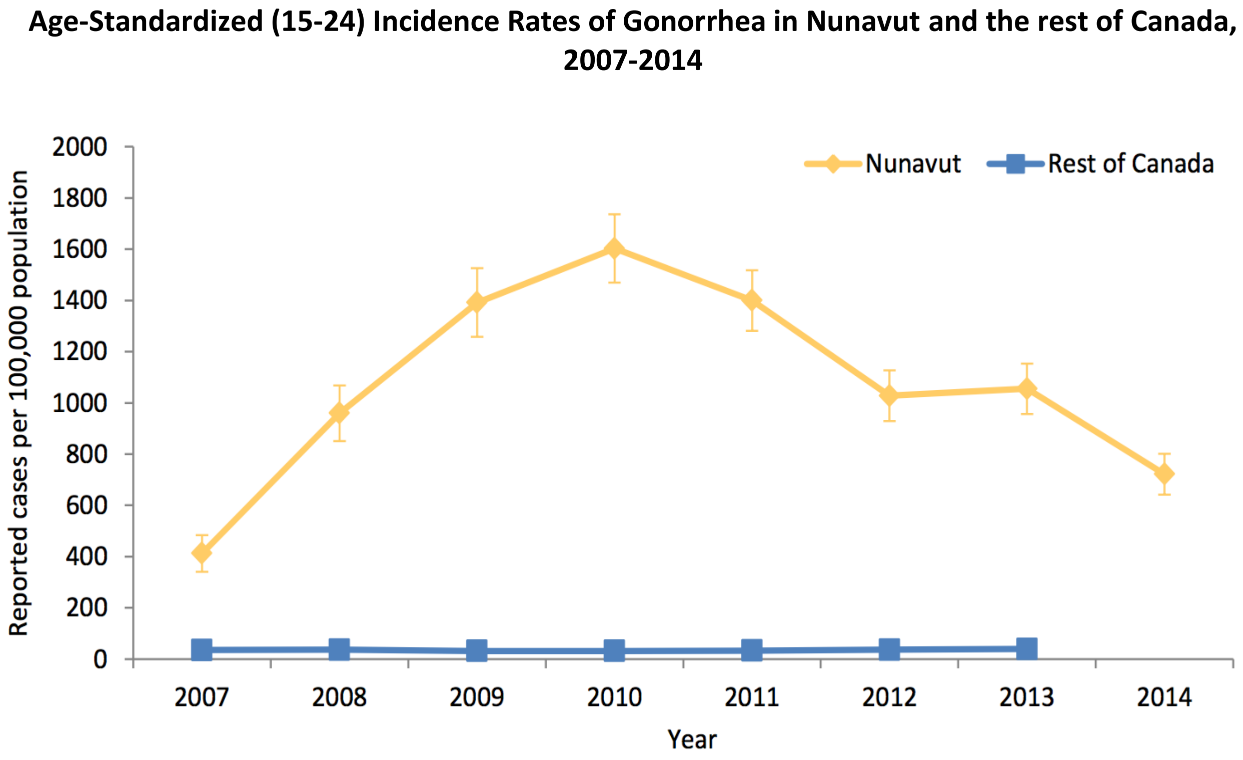

Figure 4 (1, p12).

Average Annual Crude Incidence Rates of Syphilis in Nunavut by Age and Sex, 2007 to 2014

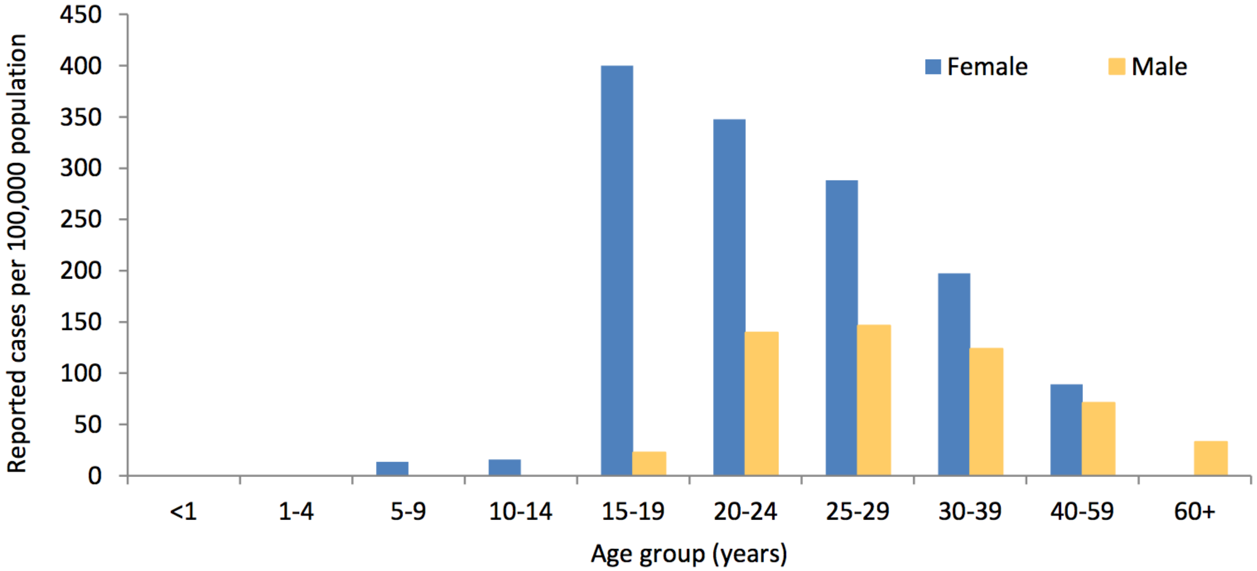

Figure 5 (1, p15)

Appendix 2: Maps

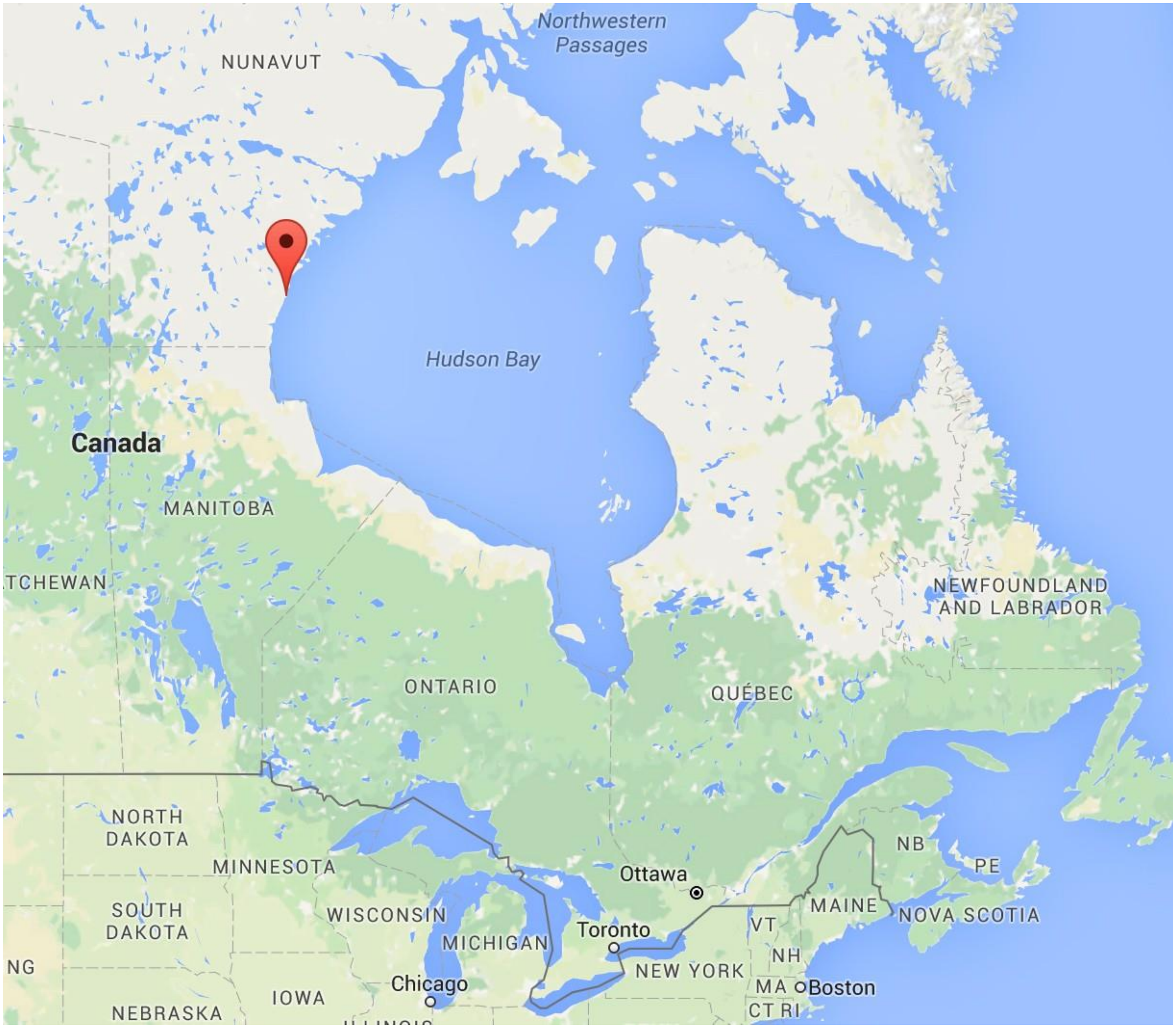

Figure 6, Arviat (at pinpoint), Nunavut, Canada (From Google Maps).
